# Importance of Linear Combination Modeling for Quantification of Glutathione and γ-Aminobutyric Acid Levels Using Hadamard-Edited Magnetic Resonance Spectroscopy

**DOI:** 10.3389/fpsyt.2022.872403

**Published:** 2022-04-25

**Authors:** Yulu Song, Helge J. Zöllner, Steve C. N. Hui, Kathleen Hupfeld, Georg Oeltzschner, James J. Prisciandaro, Richard Edden

**Affiliations:** ^1^Russell H. Morgan Department of Radiology and Radiological Science, The Johns Hopkins University School of Medicine, Baltimore, MD, United States; ^2^F.M. Kirby Research Center for Functional Brain Imaging, Kennedy Krieger Institute, Baltimore, MD, United States; ^3^Department of Psychiatry and Behavioral Sciences, Addiction Sciences Division, Center for Biomedical Imaging, Medical University of South Carolina, Charleston, SC, United States

**Keywords:** glutathione (GSH), γ-aminobutyric acid (GABA), linear combination modeling, Osprey, Hadamard-edited MRS, Gaussian, Gannet

## Abstract

**Background:**

*J*-difference-edited ^1^H-MR spectra require modeling to quantify signals of low-concentration metabolites. Two main approaches are used for this spectral modeling: simple peak fitting and linear combination modeling (LCM) with a simulated basis set. Recent consensus recommended LCM as the method of choice for the spectral analysis of edited data.

**Purpose:**

The aim of this study is to compare the performance of simple peak fitting and LCM in a test-retest dataset, hypothesizing that the more sophisticated LCM approach would improve quantification of Hadamard-edited data compared with simple peak fitting.

**Methods:**

A test–retest dataset was re-analyzed using Gannet (simple peak fitting) and Osprey (LCM). These data were obtained from the dorsal anterior cingulate cortex of twelve healthy volunteers, with TE = 80 ms for HERMES and TE = 120 ms for MEGA-PRESS of glutathione (GSH). Within-subject coefficients of variation (CVs) were calculated to quantify between-scan reproducibility of each metabolite estimate.

**Results:**

The reproducibility of HERMES GSH estimates was substantially improved using LCM compared to simple peak fitting, from a CV of 19.0–9.9%. For MEGA-PRESS GSH data, reproducibility was similar using LCM and simple peak fitting, with CVs of 7.3 and 8.8%. GABA + CVs from HERMES were 16.7 and 15.2%, respectively for the two models.

**Conclusion:**

LCM with simulated basis functions substantially improved the reproducibility of GSH quantification for HERMES data.

## Introduction

*J*-difference-edited ^1^H-MRS is a commonly used approach to quantify levels of low-concentration metabolites in the human brain (1), including but not limited to γ-aminobutyric acid (GABA) (2–4); N-acetyl aspartyl glutamate (NAAG) (5); glutathione (GSH) (6); ascorbate (Asc) (7); aspartate (Asp) (8, 9); phosphorylethanolamine (PE) (10), and lactate (Lac) (11). Metabolites that are amenable to detection by *J*-difference editing have coupled spin systems and MR signals that are overlapped in the *in vivo* spectrum. *J*-difference editing relies upon selective manipulation of the *J*-evolution of the spin system of the target metabolite. The efficiency of editing is therefore strongly dependent on the echo time (TE), with different spin systems having different optimal TEs for editing, e.g., GSH at TE ∼120 ms (12, 13) and GABA at TE ∼68 ms^2^ (2). The MEscher–GArwood Point RESolved Spectroscopy (MEGA-PRESS) (3) pulse sequence is limited to targeting one editing frequency per experiment (14). Recently, Hadamard Encoding and Reconstruction of MEGA-Edited Spectroscopy (HERMES) (15) has built upon MEGA-PRESS for the selective detection of multiple metabolites simultaneously.

The two most commonly edited metabolites are glutathione (GSH, the major antioxidant within the central nervous system), and GABA (the primary inhibitory neurotransmitter). As an antioxidant, GSH mitigates oxidative damage by neutralizing reactive oxygen species. Since oxidative stress plays an important role in a range of neurodegenerative processes (16), from healthy aging (17) to Parkinson’s Disease (PD) (18) and Alzheimer’s Disease (AD) (19), measuring GSH levels (14) in the aging brain is of great importance. Notably, one recent study demonstrated higher cortical GSH levels in older healthy volunteers, potentially as a compensatory upregulation response to age-related oxidative stress (20), though other studies reported lower GSH in aging for some regions (7, 21). *In vivo* detection of GABA is important for unraveling the role of inhibitory neurotransmission in healthy cortical processing (22) and understanding the role of inhibitory dysfunction in a range of psychiatric (23), neurological and neurodevelopmental disorders (24–26). Thus simultaneous detection of GSH and GABA in a single HERMES experiment is a promising approach for efficiently measuring two potential biomarkers of various disease processes (27, 28).

**FIGURE 1:**
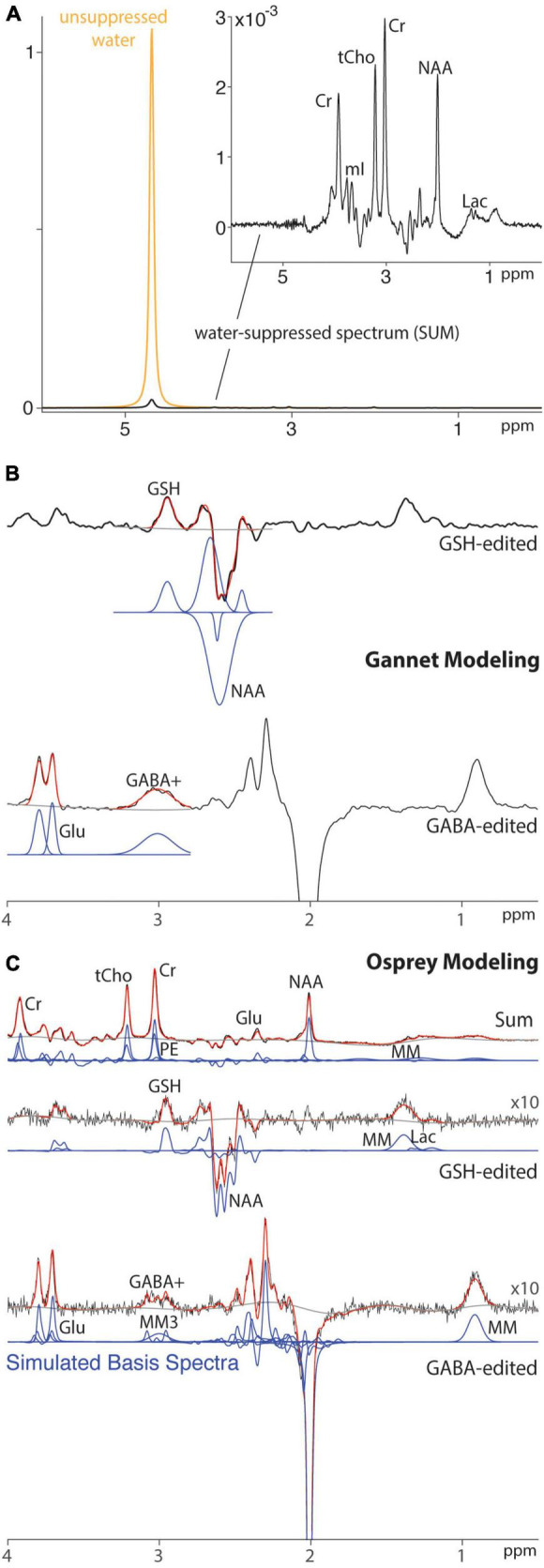
Modeling of HERMES data for a single subject using peak fitting and LCM. **(A)** Spectra acquired without (gold) and with (black) water suppression. **(B)** Peak fitting of water-suppressed HERMES data using Gannet. **(C)** LCM of water-suppressed HERMES data using Osprey. Black line: water-suppressed data. Red line: model. Gray line: baseline. Blue line: basis functions.

Magnetic resonance spectroscopy (MRS) is quantitative, in the sense that the size of signals detected is proportional to the number of spins and the concentration of molecules from which the signal derives. However, MR spectra require modeling to determine the size of signals, and in the common case of spectral overlap, to assign signal to particular metabolites. The two most commonly used approaches for modeling spectra are simple peak fitting and linear combination modeling (LCM). Simple peak fitting can be used to estimate an individual metabolite peak of interest using a simple, e.g., Gaussian (as shown in Figure 1B), lineshape model. This modeling approach is parsimonious (requiring few parameters to define the model) but has been criticized for using arbitrary model functions. Simple peak fitting is commonly used for edited spectra, which are usually relatively sparse. LCM uses a basis set to model different metabolite contributions to the full spectrum (29) (as shown in Figure 1C), thereby maximizing the prior knowledge leveraged while seeking to estimate a larger number of parameters. Recent consensus (29) recommended LCM as a best practice for quantification of edited MRS data. LCM was first applied to GSH-edited data almost 20 years ago (6), but there is relatively little literature to date that has directly compared different modeling approaches or established evidence-based best practices.

The use of simple spectral modeling in the Gannet software (30) is well-established for the quantification of GABA+-edited spectra (i.e., those in which there is a substantial macromolecular contribution mixed into the edited GABA signal) (31, 32). Although an analogous simple modeling approach has been developed for GSH, the properties of the spectrum to be modeled vary substantially with TE because of the strong TE modulation of adjacent co-edited aspartyl signals. For instance, we previously reported (33) that simple spectral modeling of HERMES data acquired at TE = 80 ms was less reproducible than simple modeling of MEGA-PRESS data acquired at TE = 120 ms. The aim of this study is to investigate whether this difference in reproducibility is indicative of a fundamental issue with multiplexed editing in HERMES, with GSH editing at TE = 80 ms, or with the modeling approach employed. We therefore compare the performance of simple peak fitting and LCM in this test–retest dataset, hypothesizing that the more sophisticated modeling approach will improve quantification of HERMES data compared with simple peak fitting.

## Materials and Methods

### Magnetic Resonance Spectroscopy Acquisition

**FIGURE 2:**
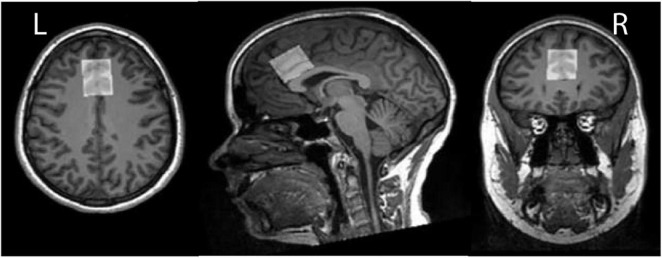
*In vivo* GSH- and GABA+-edited spectra were acquired from dorsal anterior cingulate cortex (dACC) voxel (30 mm × 25 mm × 25 mm). Here we depict the dACC voxel placement for a single exemplar subject.

A test–retest reproducibility set of HERMES and MEGA-PRESS data was previously acquired (33). Written informed consent was obtained from each participant. This study was approved by the Medical University of South Carolina Institutional Review Board. Data from twelve healthy volunteers (nine female, mean ± SD = 25 ± 2.5 years old) measured in the dorsal anterior cingulate cortex (dACC) as shown in Figure 2 were acquired with TE = 80 ms for HERMES and TE = 120 ms for MEGA-PRESS of GSH. For simplicity, these acquisitions are referred to as HERMES-80 and MEGA-120 elsewhere in the manuscript. One of the twelve participants completed only the HERMES collections so eleven subjects for all statistical analyses of the MEGA-PRESS data. Two consecutive scans were separated by a brief (5–10 min) break during which participants were removed from the scanner and then repositioned. Acquisition methods are specified fully in the original manuscript (33), and will only be briefly outlined here. Editing pulses (duration 20 ms) were applied to GABA spins at 1.9 ppm (for HERMES) and GSH spins at 4.56 ppm (for HERMES and MEGA-PRESS). Salient acquisition parameters were: TR = 2,000 ms; 256 transients; 16-step phase cycling; spectral width 2.5 kHz; 2,048 complex data points; 30 mm × 25 mm × 25 mm voxel in dACC, VAPOR water suppression (34), internal water referencing (35). HERMES editing schemes for the detection of GABA co-edit homocarnosine and macromolecular signals (15, 36); therefore, the edited 3-ppm GABA signal in the GABA-edited HERMES difference spectrum is referred to as GABA+. A matched acquisition without water suppression (as shown in Figure 1A) was acquired for eddy-current correction and quantification (35).

### Magnetic Resonance Spectroscopy Data Analysis

Simple peak fitting of the data was performed in Gannet (v3.1.5) (30)^1^ and LCM was performed in Osprey (v1.0.1.1) (37),^2^ an open-source MRS analysis toolbox. Further details about each implementation are provided below.

#### Gannet

Data processing in Gannet included spectral registration using a robust spectral registration approach for frequency-and-phase correction of the individual transients (38), weighted averaging of the transients (38), zero-filling to 32K data points, and 3-Hz exponential line broadening. The Gannet model for GABA + -edited spectra applies three Gaussian peaks to model GABA+ and the Glx [glutamate (Glu) and Glutamine (Gln)] doublet between 2.79 and 4.1 ppm, and a 4-parameter curved baseline function containing linear and quadratic terms. Gannet uses a five-Gaussian model for GSH-edited spectra acquired at TE = 80 ms, as for the HERMES data, and a six-Gaussian model for GSH-edited spectra acquired at TE = 120 ms. In both cases, a 4-parameter curved baseline function containing linear and quadratic terms data is included, data are modeled between 2.25 and 3.5 ppm, and one Gaussian is assigned to model the GSH signal and the remainder to model the complex co-edited aspartyl multiplet at ∼2.6 ppm. Water reference data were quantified with a Gaussian-Lorentzian lineshape model. Analysis of GSH and GABA+ using Gannet reproduces the values reported in our prior work (33); here we expand on this previous study by comparing these metabolite values to those estimated by Osprey (section 2.2.2).

#### Osprey

The raw data were eddy-current corrected (39) based on the water reference, and the individual transients were aligned separately within each sub-spectrum set (edit-ON or edit-OFF for MEGA-PRESS and sub-spectrum A, B, C, or D for HERMES) using robust spectral registration (38). The averaged GSH MEGA-PRESS edit-ON and edit-OFF spectra were aligned by optimizing the relative frequency and phase such that the tNAA signal at 2 ppm in the difference spectrum was minimized. The HERMES sub-spectra were aligned in three pairwise steps, adjusting the frequency and phase such that the signal in different target regions is minimized in the difference spectrum: the residual water is minimized for the GSH-OFF sub-spectra (aligning B and D), the 2-ppm tNAA signal is subsequently minimized to align the GSH-ON-GABA-OFF sub-spectrum C to D, and finally the 3.2-ppm tCho is minimized to align sub-spectrum A to C. The final GSH-edited difference spectrum is generated by subtraction (MEGA-PRESS) or Hadamard combination (HERMES). A Hankel singular value decomposition (HSVD) filter (40) was applied to remove residual water signals and to reduce baseline roll.

The basis sets were generated from a localized 2D density-matrix simulation (101 × 101 spatial grid, voxel size 30 mm × 30 mm, field of view 45 mm × 45 mm) implemented in a MATLAB-based toolbox FID-A (41), using vendor-specific refocusing pulse shape and duration, sequence timings, and phase cycling. Nineteen metabolite basis functions were included (Asc, Asp, creatine, negative creatine methylene, GABA, glycerophosphocholine, GSH, Gln, Glu, water, myo-inositol, Lac, NAA, NAAG, phosphocholine, phosphocreatine, PE, scyllo-inositol, and taurine). Spectra were modeled between 0.5 and 4.2 ppm. For the GSH-edited difference spectra, co-edited macromolecule (MM) peaks were parametrized as Gaussian basis functions at 1.2 and 1.4 ppm for MEGA-PRESS and HERMES. Similarly, two MM basis functions were added at 0.93 and 3 ppm for the GABA+-edited HERMES difference spectrum. Values of GABA+ that are reported combine the signals modeled by the GABA basis function and the MM_3_._0_ function. Amplitude ratio soft constraints are imposed on the MM and lipid amplitudes, as well as selected pairs of metabolite amplitudes, as defined in the LCModel manual (42). Osprey’s default baseline knot spacing of 0.4 ppm was used for the spline baseline.

The water-reference data were quantified with a simulated water basis function in the frequency domain with a six-parameter model (amplitude, zero-and first-order phase, Gaussian and Lorentzian line broadening, and frequency shift).

#### Quantification

Metabolite estimates for GSH and GABA+ were quantified with respect to the unsuppressed water scan. No further relaxation or tissue-segmentation corrections were employed.

### Statistical Analysis

Within-subject coefficient of variations (CVs) were calculated to quantify between-scan reliability of each metabolite (43). Bland–Altman plots (44) were used to visualize the agreement between metabolite levels obtained from the two consecutive scans. Three separate paired F-tests were conducted in R (var.test) to test for differences in variance between each modeling method (i.e., Gannet versus Osprey) for the metabolite level estimates (MEGA-120 GSH, HERMES-80 GSH, and HERMES-80 GABA+).

## Results

**FIGURE 3:**
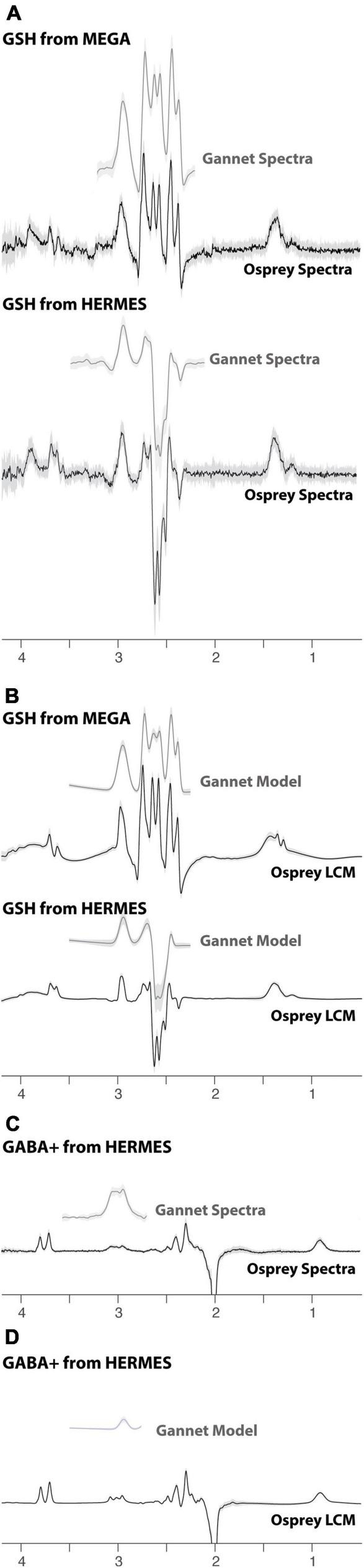
Edited difference spectra and models, using Gannet (gray) and Osprey (black) software. **(A)** Summary of GSH-edited data. **(B)** Summary of GSH models. **(C)** Summary of GABA+-edited data. **(D)** Summary of GABA+ models. The solid lines represent the group mean, and the shaded area represents the range of mean ± one SD.

All data were successfully analyzed in Gannet and Osprey. The models in each case are presented in Figure 3. It is worth noting the greater complexity of the LCM as well as the substantially different shape of the GSH model at 2.95 ppm and the different baseline handling. The variability in the models, visualized as the shaded region, is similar for Gannet and Osprey in quantifying MEGA data acquired at TE 120 ms. However, for HERMES data, LCM in Osprey appears to be less variable than Gannet with simple peak fitting.

**FIGURE 4:**
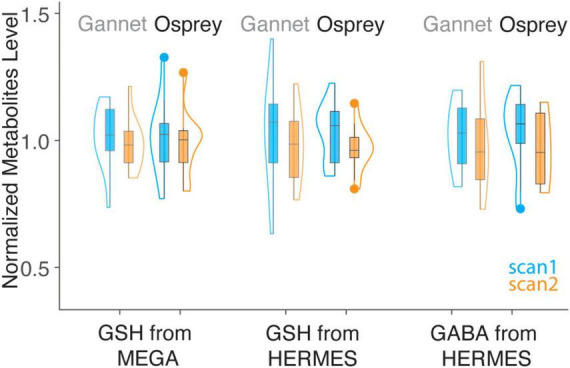
Violin plot of GSH and GABA+ estimates from simple peak fitting in Gannet and LCM in Osprey, grouped by sequence. Values for Gannet (*n* = 12 for HERMES, *n* = 11 for MEGA) and Osprey (*n* = 12 for HERMES, *n* = 11 for MEGA) have been normalized so that the mean of all values is 1, for the purposes of overlay. Note the greater variance of GSH_HERMES data using Gannet compared to Osprey (pairwise *F*-tests were used to assess the variance difference between methods).

**FIGURE 5:**
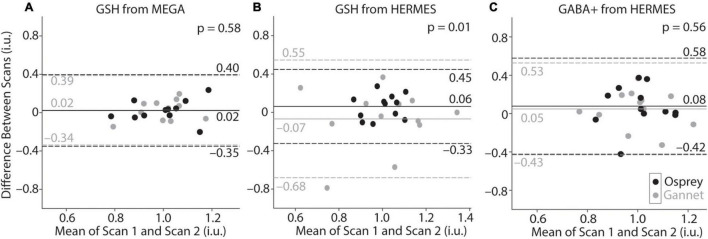
Bland-Altman plots of GSH-edited MEGA-PRESS **(A)** and GSH-edited **(B)** and GABA+-edited HERMES **(C)** data processed by Osprey (black) and Gannet (gray). Values for Gannet and Osprey have been normalized so that the mean of all values is 1, for the purposes of overlay. Solid lines represent the mean of the difference between scans, while dotted lines represent the 95% confidence interval.

Metabolite concentrations are presented in Table 1. Group-average MEGA-120 GSH levels were 2.64 ± 0.36 i.u. (quantified by Osprey) and 1.88 ± 0.21 i.u. (quantified by Gannet). Group-average HERMES-80 GSH levels were 2.94 ± 0.31 i.u. (quantified by Osprey) and 1.04 ± 0.26 i.u. (quantified by Gannet). Group-average GABA + levels were 2.87 ± 0.42 i.u. (quantified by Osprey) and 2.04 ± 0.36 i.u. (quantified by Gannet). In Figure 4, the metabolite levels are normalized to assess the variance differences between the two different algorithms. As shown in Figure 5, no differences in variance were detected between Gannet and Osprey for GSH from MEGA-120 (*F* = 0.70; *p* = 0.58, 95% confidence interval: 0.19, 2.60) or GABA+ from HERMES-80 (*F* = 1.43; *p* = 0.56, 95% confidence interval: 1.40, 16.91). However, a significant difference in variance between Gannet and Osprey was observed for GSH from HERMES-80 (*F* = 4.87; *p* = 0.01, 95% confidence interval: 0.41, 4.97).

**TABLE 1:** Metabolites levels (mean ± SD) from HERMES-80 data and MEGA-120 data processed by Osprey and Gannet.

	GSH from MEGA (i.u.) (n = 11)		GSH from HERMES (i.u.) (n = 12)		GABA + from HERMES (i.u.) (n = 12)	
	Osprey	Gannet	Osprey	Gannet	Osprey	Gannet
Scan1	2.67 ± 0.39	1.91 ± 0.24	3.03 ± 0.34	1.01 ± 0.27	2.99 ± 0.43	2.01 ± 0.29
Scan2	2.61 ± 0.33	1.85 ± 0.19	2.85 ± 0.27	1.07 ± 0.25	2.76 ± 0.41	2.08 ± 0.43
Total scan	2.64 ± 0.36	1.88 ± 0.21	2.94 ± 0.31	1.04 ± 0.26	2.87 ± 0.42	2.04 ± 0.36

*n, number of participants; i.u., Institutional Units.*

Within-subject CVs are presented in Table 2. For GSH estimates, LCM showed similar levels of reproducibility for MEGA-PRESS and HERMES data (within-subject CVs of 9.9 and 8.8%, respectively). Modeling of HERMES GSH spectra was more reproducible using LCM than simple peak fitting (within-subject CVs of 9.9 and 19.0%, respectively). For GABA + estimates, the CV of the HERMES data using Osprey was 15.2%, which is comparable to the CV of 16.7% obtained using simple peak fitting within Gannet.

**TABLE 2:** Within-subject CVs of HERMES and MEGA-PRESS data modeled by Gannet and Osprey.

		Gannet (%)(33)	Osprey (%)
GSH	MEGA-PRESS (TE = 120 ms)	7.3	8.8
	HERMES (TE = 80 ms)	19.0	9.9
GABA +	HERMES (TE = 80 ms)	16.7	15.2

*The CVs for Gannet were reported in our prior work (33).*

Bland-Altman plots are shown in Figure 5 and depict the agreement between scan 1 and scan 2 for each metabolite measurement. In Panels A and C, the distributions for Gannet and Osprey show very similar levels of variance. However, for the HERMES GSH data (Panel B), the Osprey metabolite estimates were more tightly distributed than the Gannet estimates, indicating that modeling in Osprey improved GSH reproducibility compared to Gannet.

## Discussion

The reproducibility of GSH and GABA+ metabolite concentration estimates using linear combination modeling in Osprey was compared to simple peak fitting in Gannet. The main finding was that linear combination modeling with simulated basis functions in Osprey substantially improved the reproducibility of HERMES GSH quantification, compared to the simpler Gaussian modeling implemented in Gannet. The reproducibility of quantification of GABA+ using Osprey was in good agreement with Gannet. With improved modeling in Osprey, the GSH reproducibility of HERMES-80 and MEGA-120 measurements became comparable, suggesting that HERMES is an appropriate choice for measuring GSH, with the added benefit of performing GABA editing within the same experiment.

These findings suggest that the simple modeling approach employed in Gannet is not appropriate for GSH-edited spectra acquired at TE = 80 ms. In editing GSH, frequency-selective pulses are applied to the GSH signals at 4.56 ppm in order to resolve the GSH signal at 2.95 ppm in the difference spectrum. However, these pulses also invert aspartyl signals at ∼4.5 ppm, leading to co-editing of the coupled aspartyl signals at ∼2.6 ppm. These co-edited signals are complex multiplets (two doublets of doublets) that are wide and, particularly at shorter echo times (e.g., 80 ms), have complex mixed-phase lineshapes. While the Gannet model is sufficient for modeling one metabolite of interest using a simple Gaussian lineshape, it struggles to locate the appropriate baseline for these spectra. The Osprey model uses more prior knowledge as to the expected shape of the aspartyl signals, leading to greater control of the model baseline and more reproducible quantification of GSH. In editing GABA+, the CV of the HERMES data using Osprey was 15.2%, which is comparable to the CV using simple peak fitting (33) (16.7%). A previous Gannet study (32) employed MEGA-68 to measure GABA+ levels and reported a similar CV value (16.9%), suggesting that Osprey performs comparably well when quantifying GSH and GABA+ across different TEs.

In contrast, the aspartyl signals in MEGA-120 data are more consistently phased. The choice of TE in edited experiments depends on a number of factors: longer TEs can accommodate longer, more frequency-selective refocusing pulses (45); shorter TEs suffer from less transverse relaxation; and the efficiency of editing is strongly dependent on the spin system of interest and the TE. MEGA-PRESS, in detecting one metabolite per experiment, offers greater flexibility in TE choice than HERMES, in which more than one metabolite is edited at the same TE value. In general, triplet-like signals such as GABA edit efficiently at medium TE ∼70 ms, whereas doublet-like signals such as GSH edit efficiently at long TE ∼140 ms. Importantly, when considering a TE-compromise for HERMES, triplet-like signals approach zero editing efficiency at these longer TEs. The optimal TE for editing GSH in phantoms is ∼120 ms, although owing to T_2_ relaxation, the *in vivo* edited signal is not substantially different between TEs of 68 and 120 ms (13). However, the edited spectra are substantially different, largely due to the differing behavior of the aspartyl signals. It has previously been noted (12) that this leads to spectra that are more challenging to quantify, and indeed pioneering work applying linear combination modeling to edited spectra (6) focused on GSH- and Asc-edited spectra. Medium-TE GSH editing offers signals that are less heavily T_2_-weighted, which is itself important for quantification, particularly in studies where relaxation rates may differ across comparison conditions [e.g., aging (46)]. Linear combination modeling is more robust to complex signal lineshapes than simpler modeling.

This dataset has a number of inherent limitations. In comparing MEGA-120 and HERMES-80, two factors are changing (both MEGA/HERMES and the TE) making interpretation of the results more complex. Our interpretation of the data tends to focus on the TE difference, but it is also possible that there are inherent differences between HERMES and MEGA. Additionally, this study compared two widely used models of edited MRS spectra: simple peak fitting and linear combination model with simulated basis set. We could have included additional algorithms to increase the understanding of different modeling methods. However, those comparisons would be overwhelming and beyond the scope of a single study. Thirdly, it is notable that the within-subject CV of GABA was higher than seen on average for between-subject CVs in a recent multi-site GABA+ study (47). This may reflect the limited estimation of variance in a single study with a low sample size (and these values are within the range of prior work), but also the different acquisition parameters. Compared to TE 68 ms, TE-80 GABA+ data have lower SNR due to slightly reduced editing efficiency and greater T_2_ relaxation of GABA and particularly MM signals. The longer editing pulses used also reduce the extent of MM co-editing, further impacting GABA+ SNR, and potentially introduce greater susceptibility to frequency instability. Any methodological changes that reduce the MM contamination to the GABA+ signal tend to negatively impact reproducibility. Fourthly, a larger cohort may provide stronger statistical power for these assessments.

## Conclusion

When using linear combination modeling, GSH levels showed comparable reproducibility between HERMES-80 and MEGA-PRESS-120. This finding differs substantially from our previous analysis of this dataset with simple peak modeling. The advantages of HERMES, in terms of acquiring edited GSH and GABA+ spectra in a single acquisition, do not come at the cost of worsened GSH reproducibility when linear combination modeling is used in place of simple peak modeling. Linear combination modeling based on prior knowledge metabolite basis spectra is important for reproducible analysis of medium-TE GSH-edited spectra.

## Data Availability Statement

The raw data supporting the conclusions of this article will be made available by the authors, without undue reservation.

## Ethics Statement

The studies involving human participants were reviewed and approved by Medical University of South Carolina Ethics Committee. The patients/participants provided their written informed consent to participate in this study.

## Author Contributions

YS drafted the initial version of this manuscript with significant contributions from HZ, JP, SH, GO, and RE. KH contributed to revision of the manuscript text and statistical analyses, with significant contributions from other co-authors. All authors have critically reviewed this manuscript and provided consent for publication.

## Conflict of Interest

The authors declare that the research was conducted in the absence of any commercial or financial relationships that could be construed as a potential conflict of interest.

## Publisher’s Note

All claims expressed in this article are solely those of the authors and do not necessarily represent those of their affiliated organizations, or those of the publisher, the editors and the reviewers. Any product that may be evaluated in this article, or claim that may be made by its manufacturer, is not guaranteed or endorsed by the publisher.

## Footnotes

1
https://github.com/richardedden/Gannet3.1/releases/tag/v3.1.5

2
https://github.com/schorschinho/osprey/releases/tag/v1.0.1.1
